# Systematic reviewers' perspectives on replication of systematic reviews: A survey

**DOI:** 10.1002/cesm.12009

**Published:** 2023-04-10

**Authors:** Phi‐Yen Nguyen, Joanne E. McKenzie, Daniel G. Hamilton, David Moher, Peter Tugwell, Fiona M. Fidler, Neal R. Haddaway, Julian P. T. Higgins, Raju Kanukula, Sathya Karunananthan, Lara J. Maxwell, Steve McDonald, Shinichi Nakagawa, David Nunan, Vivian A. Welch, Matthew J. Page

**Affiliations:** ^1^ Methods in Evidence Synthesis Unit, School of Public Health and Preventive Medicine Monash University Melbourne Australia; ^2^ MetaMelb Research Group, School of BioSciences University of Melbourne Melbourne Australia; ^3^ Faculty of Medicine, Dentistry and Health Sciences, Melbourne Medical School University of Melbourne Melbourne Australia; ^4^ Clinical Epidemiology Program, Centre for Journalology Ottawa Hospital Research Institute Ottawa Ontario Canada; ^5^ Faculty of Medicine, School of Epidemiology and Public Health University of Ottawa Ottawa Ontario Canada; ^6^ Bruyère Research Institute Ottawa Ontario Canada; ^7^ Department of Medicine, Faculty of Medicine University of Ottawa Ottawa Ontario Canada; ^8^ School of Historical and Philosophical Studies University of Melbourne Melbourne Australia; ^9^ Leibniz‐Centre for Agricultural Landscape Research (ZALF) Müncheberg Germany; ^10^ African Centre for Evidence University of Johannesburg Johannesburg South Africa; ^11^ Population Health Sciences, Bristol Medical School University of Bristol Bristol UK; ^12^ Interdisciplinary School of Health Sciences University of Ottawa Ottawa Ontario Canada; ^13^ Faculty of Medicine University of Ottawa Ottawa Ontario Canada; ^14^ Evolution and Ecology Research Centre, School of Biological, Earth and Environmental Sciences University of New South Wales Sydney Australia; ^15^ Nuffield Department of Primary Care Health Sciences, Centre for Evidence‐Based Medicine Oxford University Oxford UK

## Abstract

**Background:**

Replication is essential to the scientific method. It is unclear what systematic reviewers think about the replication of systematic reviews (SRs). Therefore, we aimed to explore systematic reviewers' perspectives on (a) the definition and importance of SR replication; (b) incentives and barriers to conducting SR replication; and (c) a checklist to guide when to replicate an SR.

**Methods:**

We searched PubMed for SRs published from January to April 2021, from which we randomly allocated 50% to this survey and 50% to another survey on data sharing in SRs. We sent an electronic survey to authors of these SRs (*n* = 4669) using Qualtrics. Quantitative responses were summarized using frequency analysis. Free‐text answers were coded using an inductive approach.

**Results:**

The response rate was 9% (*n* = 409). Most participants considered “replication of SRs” as redoing an SR (68%) or reanalyzing originally collected data (61%), using the same or similar methods. Participants also considered updating an SR, either one's own (42%) or others (43%), equivalent to replication. Most participants agreed that replication of SRs is important (89%). Although 54% of participants reported having conducted a replication of a SR, only 22% have published a replication within 5 years. Those who published a replication (*n* = 89) often found their replication supported (47%) or expanded the generalizability of the original review (51%). The most common perceived barriers to replicating SRs were difficulty publishing (75%), less prestige (65%), fewer citations (56%), and less impact on career advancement (55%) compared to conducting an original SR. A checklist to assess the need for replication was deemed useful (79%) and easy to apply in practice (69%) by participants.

**Conclusion:**

Reviewers have various perceptions of what constitutes a replication of SRs. Reviewers see replication as important and valuable but perceive several barriers to conducting replications. Institutional support should be better communicated to reviewers to address these perceptions.

## 1 INTRODUCTION

Replication is a research strategy used to investigate the credibility of research findings [1]. Replication can be done for various types of research, including systematic reviews (SRs), which synthesize the body of evidence on a particular topic and frequently underpin clinical practice guidelines [2]. Replication of SRs can support the results of an original review [3], or, when the results are not confirmed, provide insight into the reasons for this (e.g., the use of different design choices or analytic methods) [4].

Replication of research has been defined in various ways, such as the testing of the reliability of a prior research finding with different data [5], the repetition of a previous study, the extension of a previous study, or the road‐testing of a theory [6]. In the context of SRs, questions remain as to whether replication should entail re‐doing the entire review process or only the analysis of data; whether the analysis should use originally collected data or include additional data (e.g., when a review is updated), and whether the review question can be broadened or narrowed [7]. Exploring how systematic reviewers consider these questions can help inform the development of a consistent, standardized definition of SR replication. This is important for future discourse promoting replications that add value (e.g., validating an SR of public health importance), as opposed to unintentional or ill‐informed duplications.

Despite the merits of replication, replication studies are published at relatively low rates [8, 9, 10, 11, 12], even more so for replications of SRs [13]. An understanding of factors that encourage or discourage replications might inform the development of effective strategies to promote the conduct of high‐quality replications. A recent qualitative review identified several barriers and facilitators to achieving such a goal across science [14], but to our knowledge, no study has assessed which specific barriers or facilitators are relevant to systematic reviewers.

Like any SR, replicating a review requires a substantial commitment of time and resources. Therefore, replicating an SR that had been conducted and reported to high standards may be seen as redundant, or even duplicative [7, 15]. Even in topics where replication is useful, there exists a certain point beyond which the additional replication becomes research waste [15, 16]. A checklist has recently been developed to help systematic reviewers assess the need for a replication of a review, before actually conducting one [17]. This checklist should facilitate the conduct of more purposeful replication and can be a useful tool for funding agencies and journal editors when evaluating proposals for SR replications [14]. Exploring systematic reviewers' acceptance and attitudes toward this tool will be beneficial to assess its usefulness and applicability.

### 1.1 Objectives

We aimed to explore the perspectives of systematic reviewers on (a) the definition and importance of replication of SRs, (b) incentives and barriers to conducting replication of reviews, and (c) a checklist to guide when to replicate an SR.

## 2 METHODS

This study was conducted as part of the REProducibility and Replicability In Syntheses of Evidence (REPRISE) project. The REPRISE project consists of a suite of four studies that aim to investigate various aspects of transparency, reproducibility, and replicability of SRs of the effects of health, social, behavioral, and educational interventions [18]. Methods for all studies in the REPRISE project were prespecified in the published protocol [18]; all deviations are detailed in Supporting Information: File S1. In the present paper, we report the results of study 2a. In a companion paper, we report the results of study 2b, where we explored systematic reviewers' views on sharing review data, analytic code, and other materials.

### 2.1 Creation of the sampling frame

We systematically searched PubMed for SRs indexed from January 1 to April 30, 2021, using a pragmatic search strategy developed by an information specialist (S. M.): (meta‐analysis[PT] OR meta‐analysis[TI] OR systematic[sb]) AND 2021/01/01:2021/04/30[EDAT], with no language restriction. We selected this date range as it occurred relatively close to the time when we started setting up the survey. Given the survey required systematic reviewers to recall whether they shared any materials of their published SR, we wanted to keep the length of time between review publication and survey completion at a minimum. Records were imported to Endnote X9.3.3 for the removal of duplicates. We then sought the corresponding authors' names, email addresses, and affiliations using two methods of parsing data (implemented in R v.2.13). Specifically, parsing public information from PubMed online articles using the *easyPubMed* package [19], and parsing text from PDF full‐text files using the *pdftools* package [20]. If multiple corresponding authors were listed, only the first corresponding author's details were recorded. The collected information was reviewed for accuracy by one author (P.‐Y. N. or M. J. P.). We then excluded reviews that had been included in REPRISE Study 1 [21] or reviews co‐authored by any of the investigators of this study. The reason for the former exclusion criterion was to reduce the author burden since these authors will be contacted as part of another REPRISE study. If two or more reviews had the same corresponding author, we only included the most recent review. We screened all titles for correction, corrigendum, erratum, author's reply, or response related to an SR, and only included the SR in the sampling frame.

The sampling frame was initially stratified into countries in which the corresponding author was based. The order of entries within the sampling frame was then randomized using the function RAND() in Excel. Within each country stratum, we assigned the first 50% of the randomized entries to this survey (study 2a) and the other 50% to the companion survey on data sharing (study 2b).

### 2.2 Survey sampling and distribution

There were two phases of survey distribution. In phase 1, we administered the survey to a stratified random sample of 300 authors. We stratified the sample by country (based on the corresponding author's first affiliation) and used simple random sampling to select the reviews within each stratum (country). The number of reviews selected per country was proportional to the number of reviews in that country (proportional allocation) [22]. All random selection was performed using the RAND() function in Excel. In phase 2, we administered the survey to all remaining participants in the sample.

We took a two‐phased approach for a number of reasons. First, a random sample of 300 participants with a high response rate would provide sufficient precision and generalizable results to the population of systematic reviewers. However, we anticipated that the response rate might be low based on similar surveys (e.g., 15%), necessitating the need to increase our sample size to obtain a greater range of viewpoints (recognizing the diminishing generalizability). Second, we wanted to determine how many emails were unsuccessfully delivered, and finally, how effective our schedule of reminders was for attaining responses.

Both phases of the survey were delivered online using the survey platform Qualtrics [23]. Phase 1 commenced on July 27, 2021, whereby an invitation was sent to the 300 randomly selected authors (Supporting Information: File S2). This invitation was followed by up to three reminders, sent at 3‐week intervals, in the case of nonresponse. Given the low response rate observed in phase 1, we commenced phase 2 on October 14, 2021, with all remaining authors sent an invitation and up to three reminders at 2‐week intervals in the case of nonresponse. We shortened the period between reminders to 2 weeks in phase 2 because very few responses were recorded beyond the first few days following reminder emails in phase 1. In both phases, we dealt with notifications of unsuccessful delivery by searching the institutional website of the corresponding author to seek an accurate email address; if no such email address could be found, we used the next available corresponding author email address, when available. Phase 2 of the survey distribution was closed on December 10, 2021.

### 2.3 Survey content

Before launching the survey in phase 1, we sent a preliminary version of the survey to six systematic reviewers known to the corresponding author for piloting, all of whom reviewed the preliminary version, and provided feedback on the wording and content, which we incorporated. The final version of the survey comprised two sections (Supporting Information: File S3). The first section sought respondents' opinions on what constitutes a replication of an SR, the perceived value of and resource allocation for replication, factors that might facilitate or disincentivize replication, and reasons for conducting their own replication of SRs. Items in this section were informed by those of other surveys exploring researchers' views on the replication of other study designs [14, 24, 25, 26]. We presented to participants four groups of scenarios and asked which of these could be considered as replication of SRs: (a) redoing an SR using the same or different methods as the original review, (b) redoing an SR with a narrower or broader research question, (c) reanalyzing the data included in an SR using the same or different meta‐analytic methods as the original review, and (d) updating either one's own or another team's SR with the latest data. These scenarios were presented in random order, and participants could choose more than one applicable scenario. Participants were also invited to evaluate the usefulness and ease of use of a checklist for when to replicate SRs, developed by Tugwell et al. [17]. The second section sought the participants' demographic and professional background, including country of residence; the discipline of their research; whether they identified as methodologists, statisticians, early‐career researchers, or PhD students; and their experience with conducting SRs. The survey consisted of binary (yes/no) and 7‐point Likert scales, and open‐ended questions to allow the gathering of free‐text comments.

### 2.4 Data analysis

We used descriptive statistics (frequency and percentages) to summarize the responses to all survey questions. For ease of reporting percentages in the text, we only report the cumulative percentage of positive response options for 7‐point Likert scale questions (i.e., the sum of “Somewhat agree,” “Agree,” and “Strongly agree”) for each item. Percentages of neutral and negative responses are also available in the corresponding figure and table. Percentages were calculated based on the available data for each item; we did not impute missing data. All analyses and graphs were generated in R v.4.1.2.

We used an inductive approach [27] to coding free text comments. One author (P.‐Y. N.) read each free text response and either (i) coded them using an existing code, generated from existing response options in the survey or from coding a previous respondent's questionnaire, or (ii) assigned a new code that captured the meaning and content of the text. For example, for the question of what constitutes replication, the qualitative comment “the process of using the same or very similar methods as a previous systematic review to determine whether comparable results are obtained” was coded using the existing code “Redoing a systematic review to test whether using similar (or the same) methods described in the original review produces the same findings.” Comments such as “[…] should have the same searching and inclusion frame” and “redoing a systematic review […] without change in the number of eligible studies for the reanalysis” was assigned the new code: “Redoing a review that preserves the same scope as the original”. As each subsequent free text response was read, existing codes were reviewed and revised, and new codes were added, when necessary. All codes assigned were reviewed by another author (M. J. P.) and any discrepancies were resolved via discussion. We present the frequency and percentage of each code and provide illustrative quotes for each.

## 3 RESULTS

### 3.1 Overview

**Figure 1:**
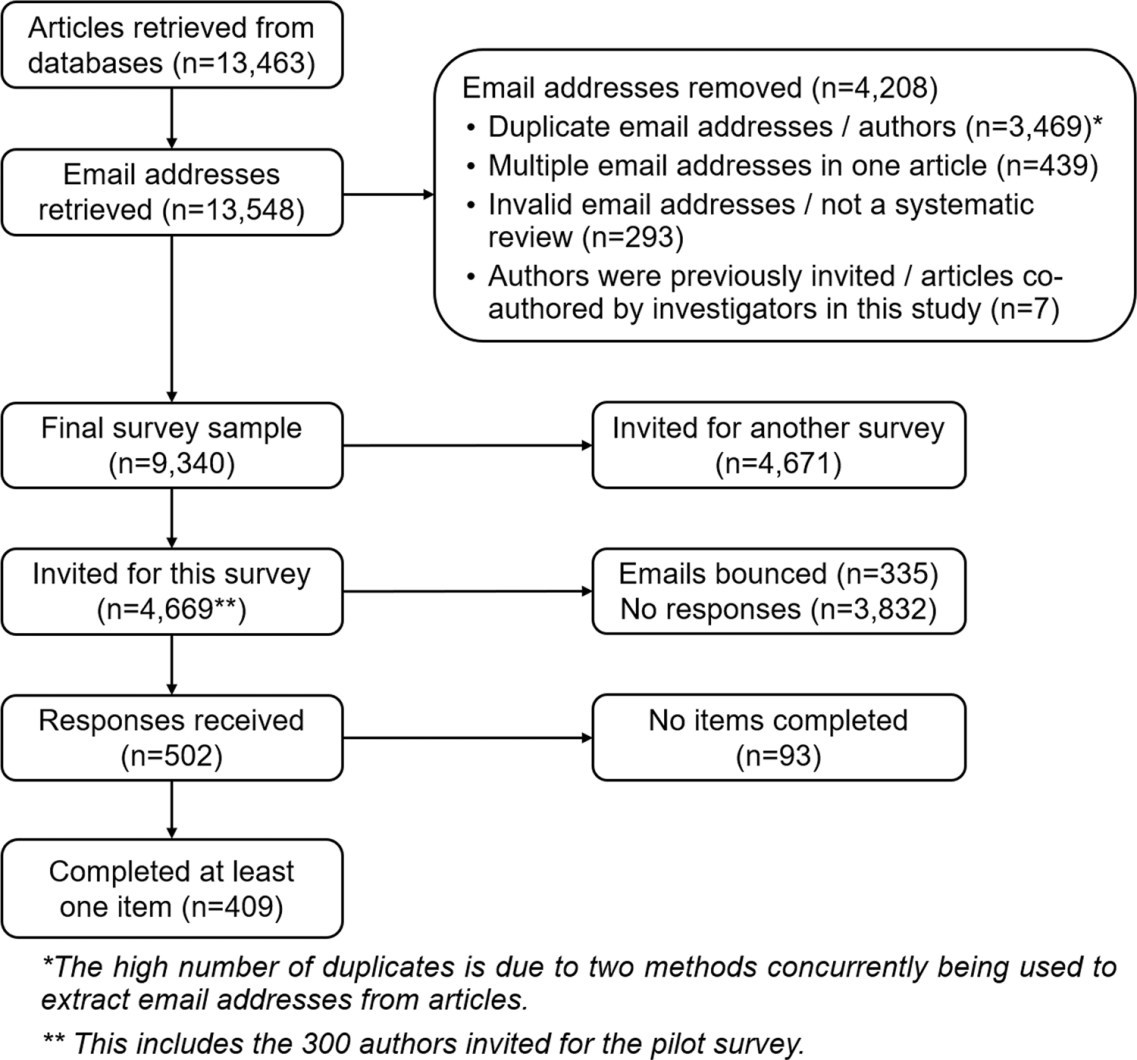
Flowchart of recruitment and data collection.

We retrieved 13,463 records from the PubMed search, from which we obtained 13,548 email addresses. After removing 4208 email addresses for various reasons (mainly because they were duplicates; Figure 1), our final sampling frame consisted of 9340 unique email addresses, of which we randomly selected 4669 (50%) for this survey. Across both phases of survey distribution, among the 4669 sent emails, 335 (7%) returned notifications of failed delivery. Of the 4334 successfully delivered emails, 502 participants consented (12%), with 409 completing at least one survey item (9%).

### 3.2 Participant characteristics

Participants were based in 55 countries. The region where most participants resided was Europe (43%), followed by the Americas (27%) and Western Pacific (19%), with the top countries being the United Kingdom (13%), the United States (11%), and Canada (10%). The majority were affiliated with a university (85%) or a hospital (30%). The most common disciplines participants worked in were medicine (46%), public health and epidemiology (21%), and psychology or psychiatry (9%). More than half the participants (56%) identified as a methodologist, while 10% identified as a statistician. Approximately 22% were PhD students and 27% had completed their PhD within the last 5 years. Almost all participants (91%) had conducted research for at least 3 years. A large percentage (74%) had co‐authored at least three SRs throughout their career. Almost all (93%) participants slightly, mostly, or completely supported the open science movement (Table 1).

**Table 1:** Participant characteristics.

	Response
	*n*/*N* (%)
*Country of residence*	
Europe	132/308 (43%)
Americas	82/308 (27%)
Western Pacific	57/308 (19%)
Eastern Mediterranean	13/308 (4%)
Africa	13/308 (4%)
South‐East Asia	11/308 (4%)
*Type of institutions affiliated with* a	
University	314/369 (85%)
Hospital	109/369 (30%)
Government department	10/369 (3%)
Commercial company	7/369 (2%)
Research institute not affiliated with any of the above	29/369 (8%)
Other institutions	8/369 (2%)
*Discipline*	
Allied health—not elsewhere specified	3/347 (1%)
Biological sciences, including genomics	9/347 (3%)
Economics	9/347 (3%)
Environmental sciences	13/347 (4%)
Epidemiology	9/347 (3%)
Medicine, including dentistry	160/347 (46%)
Nursing	25/347 (7%)
Pharmacy and pharmacology	10/347 (3%)
Psychiatry and psychology	31/347 (9%)
Public health—not elsewhere specified	63/347 (18%)
Rehabilitative allied health and sports sciences	24/347 (7%)
Social sciences and social work	4/347 (1%)
Statistics	1/347 (<1%)
Veterinary sciences	3/347 (1%)
Other	6/347 (2%)
*Job description*	
Methodologist	205/367 (56%)
Statistician	38/364 (10%)
*PhD student*	78/360 (22%)
Completed PhD within the last 5 years	97/363 (27%)
*Years of experience in research*	
Less than 3	33/367 (9%)
3–10	180/367 (49%)
More than 10	154/367 (42%)
*Number of systematic reviews co‐authored*	
1	51/367 (14%)
2	44/367 (12%)
3–10	189/367 (51%)
More than 10	83/367 (23%)
*Opinion on open science*	
Completely opposed	1/367 (<1%)
Mostly opposed	3/367 (1%)
Slightly opposed	12/367 (3%)
No opinion	11/367 (3%)
Slightly support	20/367 (5%)
Mostly support	156/367 (43%)
Completely support	164/367 (45%)

^a^ Participants could select more than one option and therefore percentages across options can add to greater than 100%.

### 3.3 Opinions on the definition of replication

**Figure 2:**
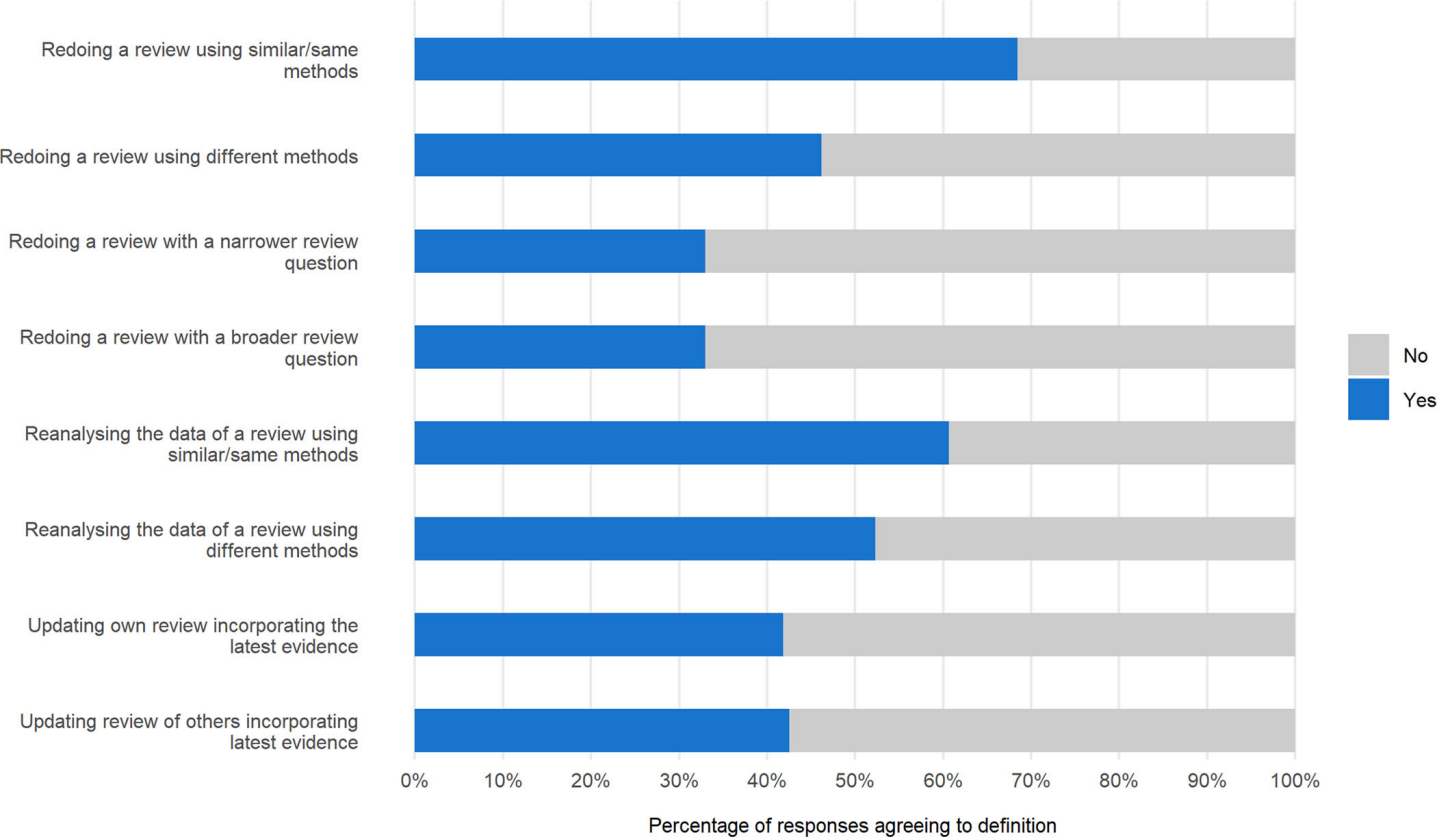
Participants' perspectives on the definition of replication.

The most accepted definitions of replication of SRs were redoing an SR (68% using the same or similar methods, 46% using different methods), and reanalyzing the data collected for a previous review (61% using the same or similar methods, 52% using different methods) (Table 2, Figure 2). Fewer participants considered updating the SR as equivalent to replication (42% for updating one's own and 43% for updating another's). Lastly, the least accepted definitions were redoing the SR with a modified research question (i.e., a change in populations, interventions, settings, or outcomes) (33% for narrower questions and 33% for broader questions).

**Table 2:** Frequency (%) of responses to each quantitative item in the survey.

	Response	
	*n*/*N* (%)	
*Perceived definition of a replication of systematic review* a		
Redoing a systematic review to test whether using similar (or the same) methods described in the original review produces the same findings	280/409 (68%)	
Redoing a systematic review to test whether using different methods for the same review question produces the same findings	189/409 (46%)	
Redoing a systematic review to test whether narrowing the original review question to a subset of populations, interventions, settings, or outcomes produces findings that are consistent with the original findings	135/409 (33%)	
Redoing a systematic review to test whether broadening the original review question to a range of populations, interventions, settings, or outcomes produces findings that are consistent with the original findings	135/409 (33%)	
Reanalyzing the data included in a previous systematic review using similar (or the same) meta‐analysis methods, to test whether the same findings are generated	248/409 (61%)	
Reanalyzing the data included in a previous systematic review using different meta‐analysis methods, to test whether the original findings are robust to variations in the analysis	214/409 (52%)	
Updating a systematic review that you had worked on previously so that it includes the latest data available to answer the review question, using similar (or the same) methods that were used in the previous version	171/409 (42%)	
Updating a systematic review that another team had worked on previously so that it includes the latest data available to answer the review question, using similar (or the same) methods that were used in the previous version	174/409 (43%)	
*Perceived importance of replication of the systematic review*		
Not important	17/406 (4%)	
Somewhat important	176/406 (43%)	
Very important	187/406 (46%)	
Unsure	26/406 (6%)	
*Perception of the amount of replication of systematic reviews within own discipline*		
There should be much less	14/377 (4%)	
There should be less	30/377 (8%)	
There should be somewhat less	27/377 (7%)	
About the right amount takes place	84/377 (22%)	
There should be somewhat more	123/377 (33%)	
There should be more	80/377 (21%)	
There should be much more	19/377 (5%)	
*Perception of use of resources for replication of systematic reviews*		
Replication of systematic reviews is not important and is a poor use of available resources	22/384 (6%)	
Replication of systematic reviews is important, but given limited resources, we should continue to focus on funding original systematic reviews	217/384 (57%)	
Replication of systematic reviews is critically important, and more of the available resources should go toward funding them	108/384 (28%)	
Unsure	37/384 (10%)	
*Frequency of conducting replication of systematic reviews*	
Never	188/408 (46%)
Rarely	138/408 (34%)
Sometimes	68/408 (17%)
Often	14/408 (3%)
Published a replication of systematic review in the last 5 years	89/405 (22%)	
*Considering the participant's latest replication of a systematic review* (*n* = 89)		
Reasons for replicating systematic review		
To test whether using similar/same methods as the original review produced the same findings	13/89 (15%)	
To test whether using different methods from the original review produced the same findings	10/89 (11%)	
To test whether narrowing the original review question produced consistent findings	11/89 (12%)	
To test whether broadening the original review question produced consistent findings	17/89 (19%)	
To incorporate new evidence that has emerged since the original review	20/89 (22%)	
Other reasons	11/89 (12%)	
Perceived contribution of the replication to the original review^a^		
The replication attempt supported the findings of the original review	42/89 (47%)	
The replication attempt extended the findings of the original review to a larger population	45/89 (51%)	
The replication attempt called into question the findings of the original review	18/89 (20%)	
The replication attempt was abandoned due to a lack of transparency in the original review	4/89 (4%)	
Other	10/89 (11%)	
*Factors that encourage or discourage replication of systematic reviews* b		
Replicated systematic reviews are likely harder to publish than original systematic reviews		
Agree	287/385 (75%)	
Disagree	45/385 (12%)	
Neutral	53/385 (14%)	
Original systematic reviews are more prestigious than replicated systematic reviews		
Agree	249/385 (65%)	
Disagree	72/385 (19%)	
Neutral	64/385 (17%)	
Original systematic reviews likely obtain more citations than replicated systematic reviews		
Agree	213/382 (56%)	
Disagree	70/382 (18%)	
Neutral	99/382 (26%)	
To get promoted/tenure, a researcher needs to publish more original than replicated systematic reviews		
Agree	213/384 (55%)	
Disagree	71/384 (18%)	
Neutral	100/384 (26%)	
It is likely easier to replicate a systematic review than it is to conduct an original systematic review		
Agree	193/384 (50%)	
Disagree	127/384 (33%)	
Neutral	64/384 (17%)	
Replication of systematic reviews would take energy and resources away from projects that reflect original thinking		
Agree	180/382 (47%)	
Disagree	138/382 (36%)	
Neutral	64/382 (17%)	
Replicating a systematic review is likely going to create conflict with the authors of the original review		
Agree	136/385 (35%)	
Disagree	132/385 (34%)	
Neutral	117/385 (30%)	
Replicating systematic reviews is expected within my field/discipline		
Agree	86/383 (22%)	
Disagree	184/383 (48%)	
Neutral	113/383 (30%)	
Replicated systematic reviews would likely bring more recognition and reward to their authors than an original systematic review would		
Agree	36/385 (9%)	
Disagree	249/385 (65%)	
Neutral	100/385 (26%)	
*Views on a checklist for replication of systematic review* b [17]		
The replication checklist includes the most important factors to consider when deciding whether to replicate a systematic review		
Agree	284/363 (78%)	
Disagree	19/363 (5%)	
Neutral	60/363 (17%)	
The replication checklist appears easy to apply in practice		
Agree	251/363 (69%)	
Disagree	55/363 (15%)	
Neutral	57/363 (16%)	
The replication checklist is likely to help researchers decide whether to replicate a systematic review		
Agree	286/363 (79%)	
Disagree	29/363 (8%)	
Neutral	48/363 (13%)	

^a^ Participants could select more than one option and therefore percentages across options can add to greater than 100%.

^b^ Three levels of agreement were created by aggregating the 7‐point Likert scale. Agree = somewhat agree or agree or strongly agree; disagree = somewhat disagree or disagree or strongly disagree; neutral = neutral.

Analysis of free‐text responses provided more perspectives on the definition of “replication” (Supporting Information: File S4). Participants suggested other components that need to be preserved in a replication: the scope of the research question (*n* = 4), the methods and set of included studies (*n* = 3), or other stages of the review process (search, data extraction, and evidence appraisal) (*n* = 1).

### 3.4 Participants' experience with replication of reviews

More than half of the participants (54%) reported that they have replicated an SR before, but only 22% (*n* = 89) have published a replication of an SR in the past 5 years. Among those who published a replication in the last 5 years, 15% and 11% did so to test whether using the same/similar or different methods as the original review, respectively, produced consistent findings. Others replicated the review to incorporate new evidence (22%) or to vary the original review question (31%) (Table 2). Other reasons for replication provided in the free‐text responses (Supporting Information: File S5) included producing a more rigorous review (*n* = 2), testing moderators of the original review associations (*n* = 2), and to “apply further critical analysis” (*n* = 1).

Among 89 participants who answered questions about their latest replication of a review, 47% found that the replication supported the findings of the original review while 51% extended the findings of the original review to a larger population (Table 2). Twenty percent reported that their replication called into question the findings of the original review and 5% abandoned the replication attempt due to lack of transparency in the original review. Similar opinions were observed in the free‐text responses (Supporting Information: File S6).

### 3.5 Opinions on the value of and resource allocation for replication

Most participants (89%) agreed that replication of SRs is somewhat or very important, and a majority (59%) agreed that more replication work is needed within their own discipline (Table 2). However, 57% felt that due to limited resources, priority should still be given to funding original SRs, as compared to 28% who supported more funding toward replication. A small percentage (6%) considered replication not important and poor use of available resources.

### 3.6 Incentives and barriers to replication

**Figure 3:**
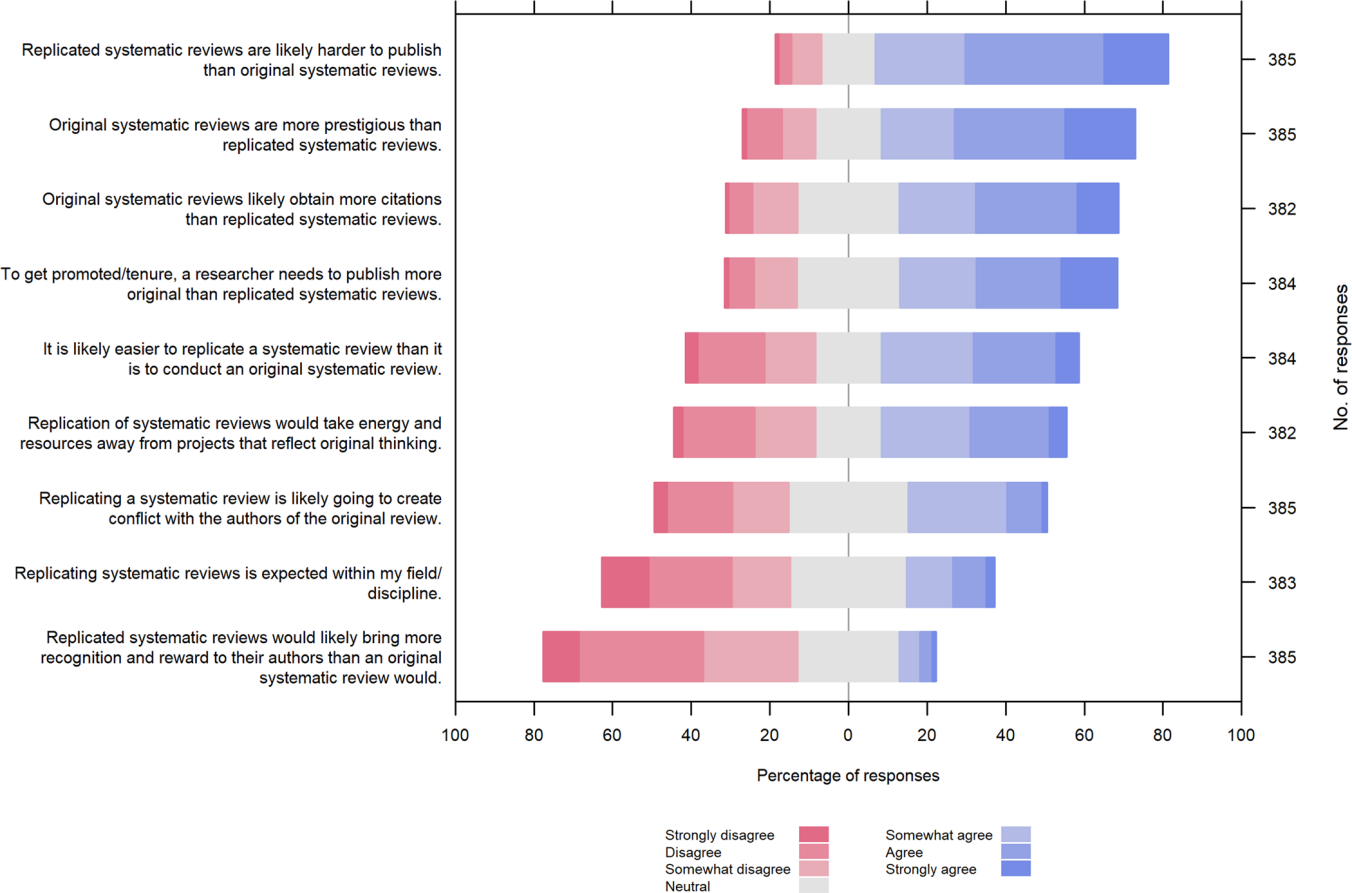
Factors that encourage or discourage replication of systematic reviews.

Factors commonly reported for discouraging replication of SRs included perceived difficulties in publishing (75% of participants agreed), less prestige (65%), fewer citations (56%), and less impact on career advancement (55%) compared with original SRs, as well as resources (time and funding) being diverted from projects that “reflect original thinking” (47%) (Table 2, Figure 3). Some participants shared the concern that replication might create conflict with the authors of the original SR (35%). On the other hand, half of the participants (50%) thought it was easier to conduct a replication than an original SR. Statements in favor of the replication of SRs received fewer agreeing responses, including that replication is expected within the discipline (22%), or that replication brings more recognition and rewards than conducting an original SR (9%).

In free‐text answers, participants also highlighted other challenges, such as difficulty in obtaining funding (*n* = 4), or difficulty in extracting the information necessary for replication due to poor reporting practices (*n* = 1) (Supporting Information: File S7).

### 3.7 Evaluation of a replication checklist

**Figure 4:**
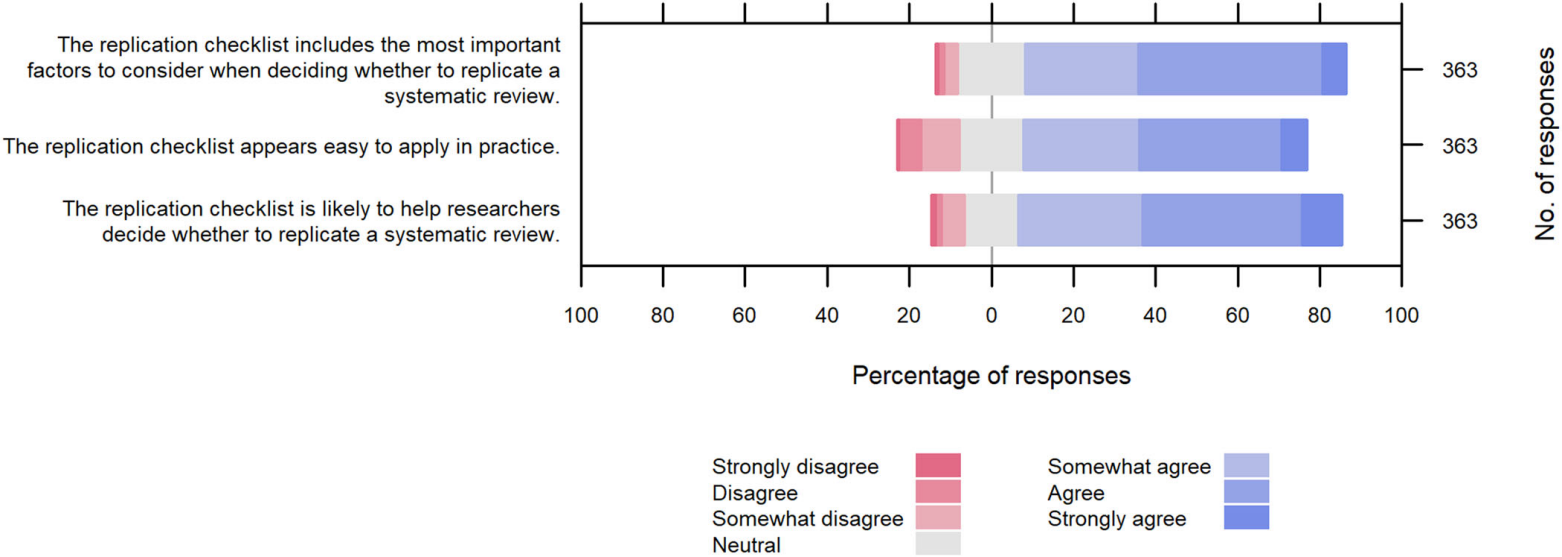
Evaluation of a checklist to decide when to replicate a systematic review by Tugwell et al. [17].

Most participants agreed that the proposed checklist to assess the need for replication [17] included the most important factors to inform when to replicate an SR (78%), and that the checklist is useful (79%) and easy to apply in practice (69%) (Table 2, Figure 4). In free‐text responses (Supporting Information: File S8), suggestions were made on ways to improve the checklist, such as defining the scope of “replication” for checklist users (*n* = 4), adapting the checklist for other types of evidence synthesis (*n* = 2), asking for justification for responses instead of just indicating yes/no (*n* = 1), and fine‐tuning the checklist based on the AMSTAR items (*n* = 1). Some concerns were raised about the potential for subjectivity and bias when completing the checklist (*n* = 4), and it was suggested that original reviewers be involved when completing the checklist (*n* = 1).

## 4 DISCUSSION

In this study, we surveyed systematic reviewers on their opinions about the replication of SRs. Despite the limited response rate, findings from the survey provide insights into how replication is perceived and practiced among participating systematic reviewers.

Our survey uncovered a range of perceptions among participants on what constitutes replication. This is perhaps not surprising, since there is no universal definition of “replication” within the systematic review community [6, 7, 13]. In this survey, the most accepted definition of replication was redoing the review using the same or similar methods, rather than just reanalyzing the original review data. This represents a strict concept of replication [7, 28] and requires reviewers to repeat several stages of the review process, namely literature search, study screening, and data collection. The second‐most accepted definition was reanalyzing the original review data using the same or similar methods. In various fields, such attempts are commonly done to evaluate the reproducibility of results and detect signs of questionable research practices, such as selective reporting or p‐hacking [1, 29]. On the other hand, fewer participants (33%) considered changing the scope of the review question (e.g., narrowing or broadening the review question) as a form of replication. Such approaches, often described as “conceptual replication,” aim to assess the generalizability of prior reviews, that is, whether the results are consistently observed in a narrower or broader range of populations, interventions, settings, or outcomes [9, 17]. Overall, varying degree of agreement about these replication definitions indicates a lack of a standardized definition for the replication of SRs. This confounds discussions about replication, since partakers may have different ideas of what replication entails, what purpose the replication serves, and the resources required for the replication. This is not unique to SRs: the lack of consensus or universal agreement on different types of replication is an issue that plagues many disciplines [6, 30]. While there have been attempts to define and classify replication in science in general [6, 30, 31], consensus is needed on what constitutes purposeful replication in SRs, how to distinguish it from ill‐informed, duplicative reviews, and whether replication should encompass updating of reviews.

Most participants recognized the value of SR replication and see replication as a good use of resources. Moreover, many participants were convinced that not enough replication was being conducted, echoing similar conclusions from opinion surveys of researchers using other study designs [24, 25], as well as the actual occurrence of replication studies in the literature [10, 11, 12]. Consistent with this sentiment is the fact that 46% of our participants reported having never conducted a replication of a review themselves. This percentage is lower than another survey, which found up to 78% of researchers had never published a replication study [24]. This discrepancy, however, might be due to our participants holding different views of what constitutes replication of an SR.

There exist several perceived barriers that discourage the conduct of SR replication. Among our participants, the main challenge was identified to be the lack of career opportunities, recognition, and rewards, rather than technical or practical difficulties in conducting them. Many participants noted a lack of expectation within their disciplines to replicate reviews. This can be attributed to the persistent belief that replication is seen as “non‐original” and not generating new evidence, and thus less valued than original reviews [25]. This sentiment has been widely documented among researchers, funding agencies, and journal editors, regardless of the type of research being replicated [24, 25, 31, 32, 33, 34]. Furthermore, this belief tends to favor replication studies that generate new findings or reconcile conflicting results but discriminates against replication studies with confirmatory results [8, 29, 35]. Lastly, since replication requires redoing the analyses and sometimes the search, screening, and data extraction, it can be impeded by the unavailability of review data and analytic code used in the original review [21, 36, 37].

There are arguments that these perceptions do not necessarily reflect the current scientific environment and the existing opportunities for researchers who conduct replication studies. In the past decade, there has been gradual progress in institutional support for replication. Funding initiatives, though limited in numbers, have been effective in promoting replication in social research [38, 39]. A recent survey found that 69%–96% of journal editors were supportive of publishing replications, illustrating a shift in sentiment compared to surveys conducted earlier [35]. Editors have also taken supportive actions, including inviting submissions of replications [29, 38], accepting replications as original research, and being transparent about editorial policies regarding replications [40]. Lastly, incentives implemented by research institutions such as training replication methods, endorsing replication studies as graduate research projects [41, 42, 43], and recognizing high‐quality replication for professional advancement [44], might also accelerate the normalization of replication in research. Moreover, recent evidence suggests that some common beliefs about replication may be untrue. For example, research that found replications had fewer citations may not have accounted for the fact that the original studies had more time to accumulate citations, and studies targeted for replication tended to be highly cited in the first place [38]. There is also no evidence that replications with contradictory results affect the citation of the original review [8, 45, 46], so replication should not be seen as unfavorable to the original review's authors.

Therefore, besides advocating for greater institutional support, it is equally important that recent progress in support for replication is better communicated to researchers, especially early‐career researchers, to address their concerns. Funding agencies should bring more attention to projects that qualify as a replication, even if the recipients did not explicitly state their intent to replicate [47]. Journal editors should refrain from editorial language that suggests bias against particular types of replication studies, such as expressing a preference for replications that offer “new insights” or “novel methods” [35], as doing so will perpetuate the negative perception of “neutral” or “null” replications as a substandard form of research.

The checklist introduced in this survey [17] was developed to facilitate the conduct of purposeful replication. Replicating an SR often takes as much time and effort as conducting a review itself. Hence, it is important that resources are directed toward replicating reviews when limited replications exist, when the topic of the review is highly relevant to public health policies, or when there are concerns about the quality or results of existing reviews. The checklist was designed as a decision aid, which authors can use to judge whether replication is necessary, before embarking on the replication itself. Journals and funding agencies can also use this tool to estimate the potential impact of a replication and guide the allocation of scarce resources (i.e., peer reviewers or funding) toward SRs most in need of replication [48]. This survey provides initial evidence of acceptability and user preferences, which can be used for further development of the checklist. The issue of what constitutes “replication” was again raised by the participants when evaluating the checklist. Providing a more salient definition of “replication” for checklist users could potentially improve its inter‐rater reliability.

There are several strengths of our study. To our knowledge, our survey is the first to explore perceptions and opinions about the replication of SRs, and how replication is defined in the specific context of an SR. The sampling frame was designed to capture systematic reviewers regardless of the area of research and geographic boundary. When asking authors which types of activities they considered replication of an SR, we presented scenarios in random order, so as to minimize bias in responses. In addition to quantitative questions, we also used open‐ended questions to help further explain the quantitative responses. Two authors were involved in checking participant email addresses for accuracy and coding of free‐text responses.

Nonetheless, our study was not without its limitations. The survey was written in English and so participants who primarily spoke another language may have felt less inclined to respond or may have misinterpreted some of the survey questions [49]. Although we took measures to minimize nonresponse, including strategies to avoid spam filter triggers (e.g., avoiding the term “survey” in the email subject heading, trialing the survey distribution), keeping the survey relatively short (∼15 min), and following up with nonrespondents [50], our response rate was low (9%). Therefore, we have refrained from reporting confidence intervals, since the validity of the confidence intervals, which is dependent on the representativeness of our sample, could be compromised by the low response rate even in the presence of random sampling. Without demographic information of nonresponders, we cannot assess the extent of nonresponse bias in our sample. There was some indication that our sample was not representative of the distribution of reviewers across countries. For example, while 12% of reviewers in the sampling frame were based in China, only 1.5% were based in China in our sample. Moreover, 56% of our responders identified as methodologists, who may have different perspectives on data sharing to systematic reviewers not doing methodological research. Therefore, our findings may not be representative of all systematic reviewers.

## 5 CONCLUSION

Systematic reviewers have diverse perceptions of what constitutes a replication. Further work on conceptualizing and defining typologies of replication of SRs is needed. Reviewers see replication as important and valuable but perceive several barriers to conducting replications. Institutional support and opportunities for replication from funding agencies, journals, and research institutions should be better communicated to systematic reviewers to address these perceptions. A checklist to evaluate the need for replication was well‐received by survey participants.

## AUTHOR CONTRIBUTIONS

**Phi‐Yen Nguyen**: Data curation, formal analysis, investigation, methodology, writing—original draft preparation. **Joanne E. McKenzie**: Conceptualization, methodology, supervision, writing—review and editing. **Daniel G. Hamilton, David Moher, Peter Tugwell, Fiona M. Fidler, Neal R. Haddaway, Julian P. T. Higgins, Raju Kanukula, Sathya Karunananthan, Lara J. Maxwell, Steve McDonald, Shinichi Nakagawa, David Nunan, Vivian A. Welch**: Writing—review and editing. **Matthew J. Page**: Conceptualization, funding acquisition, methodology, supervision, data curation, validation, writing—review and editing. The corresponding author attests that all listed authors meet authorship criteria and that no others meeting the criteria have been omitted.

## CONFLICT OF INTEREST STATEMENT

The authors declare no conflict of interest.

## ETHICS STATEMENT

This study was approved by Monash University Human Research Ethics Committee (Institutional Review Board protocol no. 28029). All participants provided their informed consent to take part in the survey at the start of the survey questionnaire. All responses were deidentified and anonymized using unique identification numbers.

## Supporting information

Supporting information.

Supporting information.

Supporting information.

## Data Availability

All data and analytic code used to generate the findings in this study is publicly accessible at osf.io/hzf7b.
