# Supplementary material for: Systematic reviewers' perspectives on replication of systematic reviews: A survey
Source: Cochrane Evid Synth Methods. 2023 Apr 10;1(2):e12009. doi: 10.1002/cesm.12009 (PMC11795895; doi:10.1002/cesm.12009)
Supplement: Supplementary file 3 — Supporting information. [file CESM-1-e12009-s003.docx]

**Title page**

S1. Deviations from the REPRISE project protocol 2

S2. Invitation email template 4

S3. Survey form 5

S4. Classifications and illustrative quotes for any other scenarios respondents thought represent a replication of a systematic review 11

S5. Classifications and illustrative quotes for any other reasons respondents provided for performing a replication of a systematic review 13

S6. Classifications and illustrative quotes for any other ways the replication attempt conducted by respondents contributed to the research question 14

S7. Classifications and illustrative quotes for any other comments respondents had about factors that can incentivise or discourage replication research 15

S8. Classifications and illustrative quotes for any other comments respondents had about the Tugwell 2020 checklist to help researchers decide whether to replicate a systematic review 16

**Supplementary File S1. Deviations from the REPRISE project protocol**

| **Original plan** | **Method implemented** | **Reason for modification** |
| --- | --- | --- |
| We would survey all systematic reviewers concurrently on (i) their views on sharing review data, analytic code and other materials and (ii) their understanding of and opinions about replication of systematic reviews. | We drafted two separate surveys addressing each of these topics and created separate equal samples of systematic reviewers for each of these surveys. | We anticipated this would help minimise respondent fatigue and improve survey completion rate. |
| We would derive the sampling frame for this study from a larger sampling frame, originally created for Study 1 of the REPRISE project. Study 1 involved systematically searching for systematic reviews of interventions indexed during a four-week period in late 2020 and evaluating their reporting characteristics. After running the searches, which yielded 6,292 unique records, we randomly sorted the records and screened 2,000 of them to meet our target sample of 300 systematic reviews. After excluding the 2,000 studies screened for REPRISE Study 1, the remaining 4,292 records constituted the sampling frame for Study 2. | We drew a new, larger sample of authors to survey using a new search strategy in PubMed: (meta-analysis[PT] OR meta-analysis[TI] OR systematic[sb]) AND 2021/01/01:2021/04/30 [EDAT] | In Study 1, 15% of the 2,000 records screened were eligible. Therefore, out of the remaining 4,292 records, we anticipated that only 640 records (15%) would meet the inclusion criteria. This left us with 320 authors to invite to complete each of the two surveys, which we considered too small a sample. |
| We would screen all remaining records using the inclusion criteria for REPRISE Study 1 and invite the corresponding authors of any eligible record (excluding those already included in Study 1). | We included all records retrieved by the new search in the sampling frame, regardless of whether they met the inclusion criteria used in REPRISE Study 1. We screened all titles to see whether the record was a correction, corrigendum, erratum, author’s reply or response related to a systematic review, and only included the systematic review in the sampling frame. | The new sampling frame was large (approximately 9,300 records), making it time-consuming to assess every record against the inclusion criteria in REPRISE Study 1. |
| We would send up to 3 reminders separated by 3 weeks. | We sent up to 3 reminders separated by 3 weeks in the pilot survey, and reduced the interval to 2 weeks in the main survey. | We needed to shorten the duration of the survey to avoid the holiday period (December to January). The pilot survey showed that most authors would have responded within the first 2 weeks. |

**Supplementary File S2. Invitation email template**

Hello,

We would like to invite you to participate in a project aiming to explore the role replication of systematic reviews plays in advancing science. Participation involves completing a survey, which will ask about your understanding of, opinions about, and previous experiences with replicating systematic reviews. The survey is designed to capture the breadth of possible perspectives on this issue and we are keen to hear from researchers such as yourself so that our findings best represent the views of those who conduct systematic reviews.

The survey takes approximately 15 minutes to complete. We anticipate that the knowledge gained will inform future methodological guidance on replication and ultimately lead to higher quality systematic reviews.

If you would like to participate, follow this link to the Survey: [link]

Or copy and paste the URL below into your internet browser: [link]

**Consent:** Providing electronic consent will be accepted as a proxy for signing a consent form, a copy of which can be downloaded here or from the first page of the survey.

You can save your responses, then close the browser and return to the survey using the same link up to one week later. The survey is compatible with mobile devices.

**Where can I get further information?**

If you would like to discuss the study, please do not hesitate to contact Matthew Page (matthew.page@monash.edu). A participant information sheet providing further information about the study can be downloaded here.

We thank you for your time and expertise.

Dr Matthew Page

Project lead and Senior Research Fellow, Monash University, Australia

On behalf of the REPRISE investigator team: Joanne McKenzie, David Moher, Fiona Fidler, Julian Higgins, Neal Haddaway, Daniel Hamilton, Raju Kanukula, Sathya Karunananthan, Lara Maxwell, Steve McDonald, Shinichi Nakagawa, Phoebe Nguyen, David Nunan, Peter Tugwell, Vivian Welch

Follow the link to opt out of future emails: [link]

**Supplementary File S3. Survey Form**

**Part A: Views on replication of systematic reviews**

| **Item** | **Response options** |
| --- | --- |
| We want to explore your perspectives on the concept of *replication*. Which of the following do you consider to be a *replication of a systematic review*? [Select all that apply]  **Scenarios were randomized.* | - Redoing a systematic review to test whether using similar (or the same) methods described in the original review produces the same findings - Redoing a systematic review to test whether using different methods for the same review question produces the same findings - Redoing a systematic review to test whether narrowing the original review question to a subset of populations, interventions, settings, or outcomes produces findings that are consistent with the original review’s findings - Redoing a systematic review to test whether broadening the original review question to a range of populations, interventions, settings, or outcomes produces findings that are consistent with the original review’s findings - Reanalysing the data included in a previous systematic review using similar (or the same) meta-analysis methods, to test whether the same findings are generated - Reanalysing the data included in a previous systematic review using different meta-analysis methods, to test whether the original findings are robust to variations in analysis - Updating a systematic review that *you* had worked on previously so that it includes the latest data available to answer the review question, using similar (or the same) methods that were used in the previous version. - Updating a systematic review that *another team* had worked on previously so that it includes the latest data available to answer the review question, using similar (or the same) methods that were used in the previous version. |
| Please specify any other scenarios that you think represent a replication of a systematic review: | Text |
| How important would you say replication of systematic reviews is? | - Not important - Somewhat important - Very important - I am not sure |
| How often do you replicate systematic reviews? | - Never - Rarely - Sometimes - Often |
| Have you published a replication of a systematic review in the last 5 years? | - Yes - No - I cannot remember |
| If yes, why did you perform the replication (if you have published more than one, consider the most recent replication only)? | - To test whether using similar (or the same) methods described in the original review produced the same findings - To test whether using different methods for the same review question produced the same findings - To test whether narrowing the original review question to a subset of populations, interventions, settings, or outcomes produced findings that were consistent with the original review’s findings - To test whether broadening the original review question to a range of populations, interventions, settings, or outcomes produced findings that were consistent with the original review’s findings - Other reason (please specify): ___________ |
| If yes, how did your replication attempt contribute to the research question (if you have published more than one, consider the most recent replication only)? [Select all that apply] | - The replication attempt supported the findings of the original review - The replication attempt extended the findings of the original review to a larger population - The replication attempt called into question the findings of the original review - The replication attempt was abandoned due to a lack of transparency in the original review - Other (please specify) |
| Do you think enough replication of systematic reviews takes place in your discipline? | - There should be much less - There should be less - There should be somewhat less - About the right amount takes place - There should be somewhat more - There should be more - There should be much more |
| Do you consider replication of systematic reviews to be a good use of resources? | - Replication of systematic reviews is not important and is a poor use of available resources - Replication of systematic reviews is important, but given limited resources we should continue to focus on funding original systematic reviews - Replication of systematic reviews is critically important, and more of the available resources should go towards funding them - I do not know |
| **The following questions relate to possible factors that encourage or discourage replication of systematic reviews.**  **How much do you agree with the following statements?** | |
| Replicated systematic reviews are likely harder to publish than original systematic reviews. | - Strongly disagree - Disagree - Somewhat disagree - Neutral - Somewhat agree - Agree - Strongly agree |
| It is likely easier to replicate a systematic review than it is to conduct an original systematic review. | - Strongly disagree - Disagree - Somewhat disagree - Neutral - Somewhat agree - Agree - Strongly agree |
| Replicating systematic reviews is expected within my field/discipline. | - Strongly disagree - Disagree - Somewhat disagree - Neutral - Somewhat agree - Agree - Strongly agree |
| Original systematic reviews are more prestigious than replicated systematic reviews. | - Strongly disagree - Disagree - Somewhat disagree - Neutral - Somewhat agree - Agree - Strongly agree |
| Replication of systematic reviews would take energy and resources away from projects that reflect original thinking. | - Strongly disagree - Disagree - Somewhat disagree - Neutral - Somewhat agree - Agree - Strongly agree |
| Replicated systematic reviews would likely bring more recognition and reward to their authors than an original systematic review would. | - Strongly disagree - Disagree - Somewhat disagree - Neutral - Somewhat agree - Agree - Strongly agree |
| Original systematic reviews likely obtain more citations than replicated systematic reviews. | - Strongly disagree - Disagree - Somewhat disagree - Neutral - Somewhat agree - Agree - Strongly agree |
| Replicating a systematic review is likely going to create conflict with the authors of the original review | - Strongly disagree - Disagree - Somewhat disagree - Neutral - Somewhat agree - Agree - Strongly agree |
| To get promoted/tenure, a researcher needs to publish more original than replicated systematic reviews | - Strongly disagree - Disagree - Somewhat disagree - Neutral - Somewhat agree - Agree - Strongly agree |
| **In 2020, Tugwell et al. produced a consensus-based checklist to help researchers decide whether to replicate a systematic review or not (**[**https://www.bmj.com/content/370/bmj.m2864**](https://www.bmj.com/content/370/bmj.m2864)**). The replication checklist includes the following 4 questions:**   1. **Has the priority for replication been assessed as high? Yes/No** 2. **Is it likely that direct replication by repetition or conceptual replication by broadening or narrowing of the scope will address uncertainties, controversies, or the need for additional evidence related to:**    1. **The framing of the question in previous reviews? Yes/No**    2. **The conduct and reporting of previous reviews? Yes/No**    3. **Author influence or conflicts of interest in previous reviews? Yes/No**    4. **Discordant findings in previous reviews? Yes/No** 3. **Would the implementation of the findings of a replication be likely to have a potentially important sizeable individual benefit or harm or affect a sizeable population? Yes/No** 4. **Are resources (time, money) best spent on replication rather than on alternative systematic reviews (considering opportunity cost)? Yes/No**   **We are interested in your views on the replication checklist. How much do you agree with the following statements?** | |
| The replication checklist includes the most important factors to consider when deciding whether to replicate a systematic review. | - Strongly disagree - Disagree - Somewhat disagree - Neutral - Somewhat agree - Agree - Strongly agree |
| The replication checklist appears easy to apply in practice. | - Strongly disagree - Disagree - Somewhat disagree - Neutral - Somewhat agree - Agree - Strongly agree |
| The replication checklist is likely to help researchers decide whether to replicate a systematic review. | - Strongly disagree - Disagree - Somewhat disagree - Neutral - Somewhat agree - Agree - Strongly agree |
| If you found any of the items in the replication checklist difficult to understand, please specify them here: | Text |
| If you think other items should be added to the replication checklist, please specify them here: | Text |
| If you have any other comments on the replication checklist which you would like to express, please record them here: | Text |
| If you have any other thoughts about replication of systematic reviews which you would like to express, please record them here: | Text |

**Part B: General characteristics**

| **Item** | **Response options** |
| --- | --- |
| What is your primary country of residence? | Drop-down list of countries |
| What type(s) of institution are you affiliated with? [Select all that apply] | - University - Hospital - Government department - Commercial company - Research institute (not part of a university, hospital, government department or commercial company) - Other (please specify) |
| What research discipline do you primarily work in (e.g. medicine, nursing, psychology, economics, environmental sciences)? | Text |
| Do you conduct methodological research on systematic reviews? | - Yes - No |
| Are you a statistician? | - Yes - No |
| Are you a PhD student? | - Yes - No |
| Did you complete your PhD within the last 5 years? | - Yes - No - Not applicable (I do not have a PhD) |
| Approximately how many years have you been doing research? | - Less than 3 years - 3 to 10 years - More than 10 years |
| Approximately how many completed systematic reviews are you a co-author of (please count published and unpublished reviews)? | - None - One - Two - Three to 10 - More than 10 |
| In general, how do you feel about open science?  By open science, we mean the movement to make scientific content (e.g. publications, data, analytic code and other research materials) publicly available with minimal barriers to access (e.g. content is able to be viewed or downloaded without payment, registration or approval). | - Completely opposed - Mostly opposed - Slightly opposed - No opinion - Slightly support - Mostly support - Completely support |

**Supplementary File S4. Classifications and illustrative quotes for any other scenarios respondents thought represent a replication of a systematic review**

| **Classification** | **Illustrative quote** | **Freq. (%)** |
| --- | --- | --- |
| Updating is different to replicating a review | “Generally speaking, I do not think that an update is a replication, as long as it includes new study. As "replication" I understand redoing a review, on the same research question, with almost the same (but also slightly different) inclusion criteria, so redoing the selection process, the data extraction and the data analysis, to see if results are the same. | 10/409 (2%) |
| Redoing a review that preserves the same scope as the original | “In my view, modifying the research question would alter the search keywords and in turn the records that would be included/excluded. Therefore, these scenarios would not count as replication.” | 4/409 (1%) |
| Redoing a review using strictly the same scope of question, methods and included studies | “My answers are all contingent on my understanding of what is meant by replication. I am not referring to updates, or SRs with slightly different questions (broader, narrower), outcomes etc... I see replication as complete identical replication” | 3/409 (1%) |
| None of the scenarios listed in the survey are examples of replicating a review | “None” | 2/409 (0.5%) |
| Redoing specifically the search, data extraction and critical appraisal | “Redoing the search, redoing the data extraction, redoing critical appraisal” | 1/409 (0.2%) |
| Redoing the review using same methods in a different context/setting (population) but same intervention and outcomes | “Redoing the SR using same methods in a different context/setting (population) but same intervention and outcomes.” | 1/409 (0.2%) |
| Redoing a review by focusing on a more specific question than the one addressed in the original review | “To be focused on more specific question, which was treated only superficially by the first one” | 1/409 (0.2%) |
| Redoing a review because questionable eligibility criteria were applied originally | “Replication is important when questionable inclusion and exclusion have been applied”.” | 1/409 (0.2%) |
| Redoing a review because the original one was deemed inadequate | “We can replicate a systematic review if the previous systematic review study had a low-quality methodologist or result, and we can improve the quality of the study by using relative advantages items.” | 1/409 (0.2%) |
| Redoing a review so it addresses upcoming challenges in the problem domain | “It must be useful to address the upcoming challenges in the problem domain and provide relevant answers to the potential research questions. It should not be the same copy as that of existing findings, rather, add something valuable to the body of knowledge.” | 1/409 (0.2%) |
| Redoing a systematic review with another team | “Redoing a systematic review with another team. Certain choices in selecting papers, data extraction or quality assessment remain slightly subjective despite clear protocols.” | 1/409 (0.2%) |
| Updating a previous systematic review (whether worked on or not), including the latest data available with different methods from the previous version | “Updating a previous systematic review (whether worked on or not), including the latest data available with different methods from the previous version” | 1/409 (0.2%) |
| Updating a previously published review while also reanalysing the data in the previous review | “Updating a systematic review in a way that involves reanalysis of the data in the previous systematic review is partly replication, partly updating. I think.” | 1/409 (0.2%) |

**Supplementary File S5. Classifications and illustrative quotes for any other reasons respondents provided for performing a replication of a systematic review**

| **Classification** | **Illustrative quote** | **Freq. (%)** |
| --- | --- | --- |
| To attempt to produce a more rigorous review | “Earlier reviews done some time ago (and weren't really systematic)” | 2/89 (2%) |
| To test moderators of the original review associations | “To test moderators of the original review associations” | 2/89 (2%) |
| To apply critical analysis | “Critical analysis” | 1/89 (1%) |
| Reason not applicable to systematic reviews | “To test whether data generated on identical populations using same/similar techniques at independent laboratories are consistent and produced similar findings” | 1/89 (1%) |

**Supplementary File S6. Classifications and illustrative quotes for any other ways the replication attempt conducted by respondents contributed to the research question**

| **Classification** | **Illustrative quote** | **Freq. (%)** |
| --- | --- | --- |
| The replication incorporated latest evidence | “The replication provided up-to-date findings about the subject matter” | 2/89 (2%) |
| The replication provided data on previously unexamined outcomes | “The replication assessed diabetes remission for the first time. All reviews to date just looked at average blood glucose markers. We also used a series of *a priori* definitions for diabetes remission as well as unpublished data from authors of RCTs. Not sure if this is technically a "replication"!” | 1/89 (1%) |
| The replication evaluated whether better assessment tools had been developed | “It was to see if better assessment tools had been developed” | 1/89 (1%) |
| The replication supported some previous findings, and called into question others | “Supported some findings, called others into question” | 1/89 (%) |

**Supplementary File S7. Classifications and illustrative quotes for any other comments respondents had about factors that can incentivise or discourage replication research**

| **Classification** | **Illustrative quote** | **Freq. (%)** |
| --- | --- | --- |
| Funding for replication research is more difficult to obtain. | “[…] peer reviewers in funding competitions will often see a proposed systematic review replication as being of lesser innovation (and perhaps lesser relevance) than other research that will explore new treatments, fill what may be considered more vital knowledge gaps, etc. Thus while replication can be important, I think from a funding perspective researchers will encounter more barriers unless other sources of funding are available.” | 4/385 (1%) |
| Replication studies are less likely to be published. | “I imagine they would be difficult to publish though unless there was significant controversy.” | 3/385 (0.8%) |
| Researchers receive less institutional support to carry out replication studies. | “Replication to confirm accuracy is useful but will be highly discouraged by senior clinicians for budding clinical academics.” | 2/385 (0.5%) |
| Poor reporting practices make it difficult to obtain accurate data for replication. | “[…] we see how difficult it is to even reproduce (not even replicate) published meta-analyses (i.e., redoing the analyses using the dataset provided by study authors or when re-extracting effect sizes), because of reporting inconsistencies, omissions, errors, and too many degrees of freedom in extracting effect sizes. | 1/385 (0.3%) |
| Fear of conflicts with the original review’s authors can discourage replication effort. | “Who dares to replicate a meta-analysis conducted by a well-recognized-in-the-field author? Not many, at least, not the ones who want to grow in the field.” | 1/385 (0.3%) |
|  |  |  |

**Supplementary File S8. Classifications and illustrative quotes for any other comments respondents had about the Tugwell 2020 checklist to help researchers decide whether to replicate a systematic review**

| **Classification** | **Illustrative quote** | **Freq. (%)** |
| --- | --- | --- |
| The checklist is useful to replicators | “I feel this checklist could be highly useful for researchers when applying for grants regarding replications of systematic reviews.” | 6/363 (2%) |
| The scope of 'replication' needs to be more clearly defined for user | “Some items seem to go quite beyond replication. For example, if you broaden or narrow the scope of an existing review, I can imagine you could find different results (that just apply on a different level). Such information is important, but does not really relate to replicability in my point of view.” | 4/363 (1%) |
| There is potential for subjectivity and bias when completing the checklist | “It seems like the checklist would lend itself to confirmation bias. If you had a notion that a review ought to be replicated for whatever reason, you could probably find a way to check off enough items.” | 4/363 (1%) |
| The checklist is unnecessary | “To me, it seems reasonable that all systematic reviews should be replicated, while acknowledging that this will not always be feasible. With that in mind, the only criterion for whether to undertake a replication review would be whether a replication has already been undertaken.” | 3/363 (0.8%) |
| Checklist should be modified to apply to other types of evidence synthesis | “Items need to be modified/included to apply to systematic review updates. SR updates are equally important as SR replication to verify the findings.” | 2/363 (0.6%) |
| AMSTAR items could be used to fine-tune this checklist | “Maybe some aspects of the AMSTAR system could be used to fine-tune this checklist but it looks overall OK to me.” | 1/363 (0.3%) |
| Replicators should collaborate with original reviewers when deciding to replicate | “Maybe consider cooperating with the authors from the review you are trying to replicate?” | 1/363 (0.3%) |
| Users should be asked to provide justifications rather than just answering yes/no | “Personally, I dislike the simple yes/no answer format. I think it would be more valuable to ask the respondent to elaborate on their answers and decisions. I believe this to be important for both the researcher (can help in motivating the introduction) and reviewers/readers to understand the reasoning for replicating a given review.” | 1/363 (0.3%) |
